# Endotracheal Tube Cuff Pressures in the Operating Room of a Pediatric Hospital: A Quality Improvement Initiative

**DOI:** 10.1097/pq9.0000000000000619

**Published:** 2022-12-07

**Authors:** Kelly M. Moon, Sherry Donaworth, Molly S. Hagele, Stephani S. Kim, Brittany L. Willer, Joseph D. Tobias

**Affiliations:** From the * College of Nursing, University of Cincinnati, Cincinnati, Ohio; † Department of Anesthesiology & Pain Medicine, Nationwide Children’s Hospital, Columbus, Ohio; ‡ Department of Anesthesiology & Pain Medicine, The Ohio State University College of Medicine, Columbus, Ohio.

## Abstract

Endotracheal tube (ETT) cuff pressures are frequently out of the recommended range of 20–30 cm H_2_O. This can lead to multiple iatrogenic complications ranging from cough, sore throat, and tracheal edema to more serious medical issues such as tracheal stenosis, aspiration, nerve injuries, and tracheal rupture. Additionally, current methods to inflate ETT cuffs vary between anesthesia providers and are not consistent in the recommended range. The objective of this quality improvement project was to increase the percentage of cuff pressures between 20 and 30 cm H_2_O.

**Methods:**

Four plan-do-study-act (PDSA) cycles were completed in the operating rooms at Nationwide Children’s Hospital over 9 months to assess ways to improve the accuracy of obtaining recommended ETT cuff pressures. Control charts were used to evaluate the primary outcome measure.

**Results:**

Preimplementation, ETT cuff pressures were out of the recommended range 76% of the time. Cuff pressures were out of the recommended range 64% of the time with the addition of the air method, 84% of the time in the tidal volume ratio cycle, and 50% of the time using the removal of air technique. The removal of air method was the most effective in achieving cuff pressures within the recommended pressure range (*P* < 0.001).

**Conclusions:**

Using quality improvement methodology, the percentage of ETT cuff pressures falling within the recommended pressure range increased using the removal of air technique. This approach is a simple and practical method that can be easily implemented in the clinical setting and would provide additional safety in the anesthetic management of pediatric patients.

## INTRODUCTION

Endotracheal intubation is a common procedure occurring approximately 15–20 million times yearly in the United States alone.^1^ There are two types of endotracheal tubes (ETTs), cuffed and uncuffed. In today’s clinical environment, anesthesia providers use cuffed ETTs most frequently.^2^ Historically, anesthesia providers used uncuffed ETT in children because of their airway size and anatomy.^12^ This teaching has recently been challenged, and cuffed ETTs are now routinely used in the pediatric setting.^2^ With the increased use of cuffed ETTs, ETT cuff pressures are increasingly important.

The benefits of using a cuffed ETT are numerous. Proper cuff inflation allows for positive pressure ventilation, administering anesthetic gases, positive end-expiratory pressure, decreased risk of airway injury related to multiple laryngoscopy attempts, and protection from aspiration of gastric contents. In addition, once inflated, the cuff pressure directly correlates to the pressure exerted on the tracheal mucosa wall.^3^ The recommended range for ETT cuff pressure is 20–30 cm H_2_O.^4,5^ This narrow range ensures that the cuff pressure is high enough to seal the airway but low enough to maintain tracheal capillary perfusion pressure.

Cuff pressures are out of the recommended range 60%–80% of the time in the clinical setting.^6-14^ This statistic is particularly concerning in the pediatric population as airway complications related to cuff pressures can lead to serious patient safety concerns.^2,15,16^ Cuff overinflation is directly correlated to airway complications such as sore throat, cough, edema, and inflammation or more serious complications such as nerve injury, tracheal stenosis, tracheal fistulas, granulations, ulcerations, and tracheal rupture.^1,13,15,17^ Underinflated ETT cuffs may place the patient at risk for aspiration and inadequate ventilation.^1,13,15^ The true incidence of complications is unknown as many of these adverse outcomes go undiagnosed.

Multiple meta-analyses, randomized controlled trials, and systematic reviews have established a correlation between cuff pressure and airway complications.^10,15,18,19^ Injuries related to endotracheal intubation can potentially increase the average length of stay and readmission rates, thereby increasing healthcare costs.^20^ Iatrogenic patient complications also result in decreased patient satisfaction, an assessed measure for healthcare facilities under the Consumer Assessment of Healthcare Providers and Systems (CAHPS). Patient dissatisfaction could lead to negative financial implications for hospital systems as reimbursement incentives are closely linked to patient experience.^21^

Monitoring ETT cuff pressure with a cuff manometer is the gold standard for cuff pressure management.^11,22^ This device is rarely used in the operating room environment and has problems such as unintentional leaks, increased expense, and the need to clean the equipment between patients.^22–26^ Despite manometry being the standard, most practitioners do not use them. Instead, many anesthesia providers inflate the ETT cuff using “estimation techniques.” These methods are used 98% of the time in the clinical setting but are subjective and not consistently accurate.^7,13,14,24–26^ Also, the inflation method used is at the provider’s discretion resulting in a wide variety of methods used in the clinical setting. With objective measurements not being used, estimation techniques being unreliable, and the wide range of methods practitioners use in the clinical setting, cuff pressures continue to be inaccurate. Injuries from mismanagement of ETT cuffs are preventable. Therefore, in the interest of patient safety, there is a need for a change in anesthesia practice. This quality improvement initiative aimed to increase the percentage of ETT pressures in the recommended range of 20-30 cmH20 in patients receiving general ETT anesthesia ages birth to 16 years old, from 25% to 60% in 5 months.

## METHODS

### Context

Nationwide Children’s Hospital is a quaternary care pediatric hospital that performs around 40,000 anesthetics annually on a wide range of patients. Anesthetic care is performed in a collaborative care team environment with attending physicians, fellows, residents, certified registered nurse anesthetists, and student registered nurse anesthetists. Following endotracheal intubation, the ETT cuff is inflated. This process is completed using a subjective or “estimation” method. The most common methods used in our clinical setting consist of listening for a leak in the mouth when placed in the ventilator, palpating the pilot balloon to evaluate cuff inflation, using a stethoscope to listen for a leak at the suprasternal notch over the trachea, and palpating the ETT cuff over the trachea to assess balloon inflation. As no standard method is performed, the cuff inflation method ultimately depends on the anesthesia provider in the room.

Preimplementation data were collected to establish a baseline for ETT cuff pressures in the operating rooms. Cuff pressures were randomly measured following ETT intubation on 25 subjects. Once we discovered that our baseline data were out of the recommended range, the QI team examined opportunities for improvement.

### Interventions

**Fig. 1.**
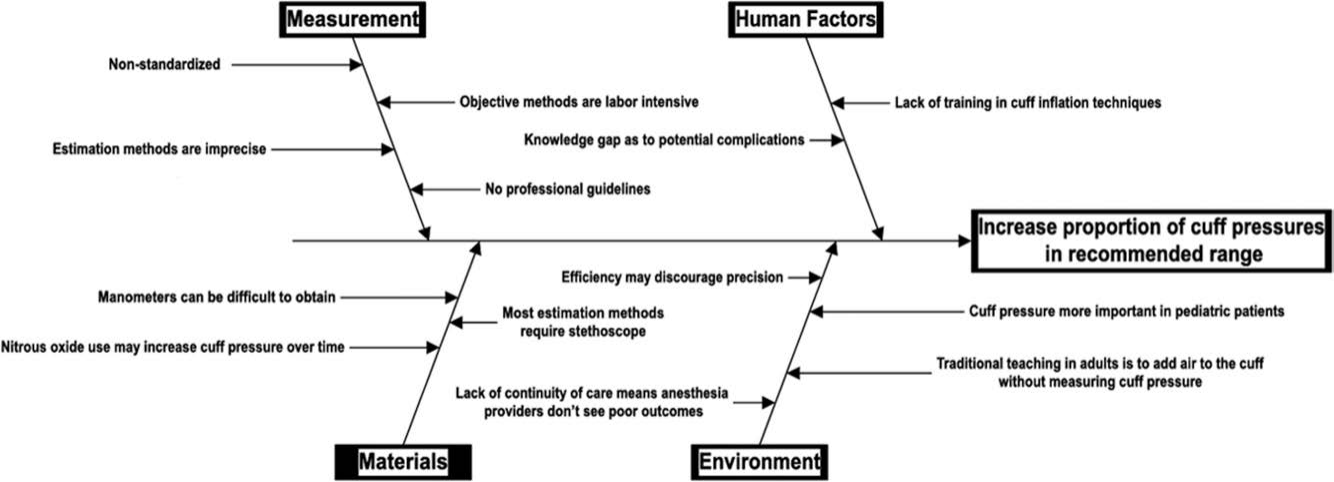
Fishbone diagram for improving ETT cuff pressures in the operating room.

**Fig. 2.**
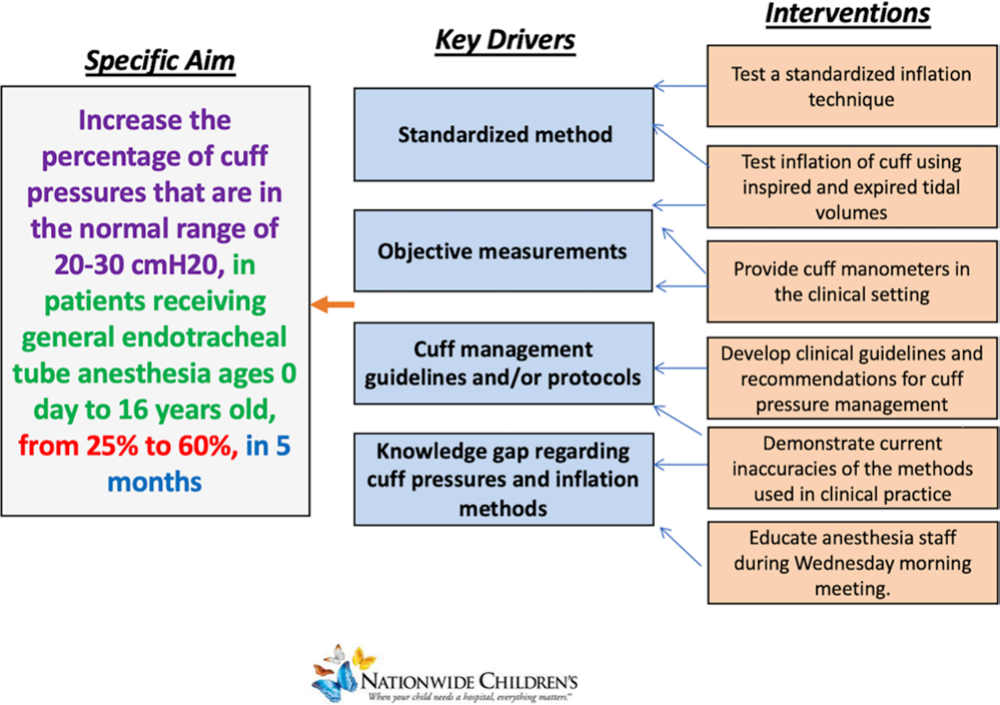
Key driver diagram for Nationwide Children’s Hospital ETT cuff pressure quality improvement project.

This manuscript adheres to the Standards for Quality Improvement Reporting Excellence (SQUIRE) 2.0. We used quality improvement science by applying the Institute for Healthcare Improvement Model for Improvement methodology. We created a fishbone diagram and key driver diagram to aid in categorizing the barriers to accurate and precise cuff pressure maintenance and implementing interventions (Figs. 1 and 2). Our key drivers consisted of standardizing a method, using objective measurements, creating cuff management guidelines, and educating staff regarding the importance of cuff pressures and cuff inflation methods (Fig. 2). This project was conducted from October 2020 to July 2021. The quality improvement project team, consisting of two nurse anesthetists and two anesthesiologists, performed cuff inflation for PDSA cycles 1, 2, and 3. The inclusion of a larger group of anesthesia providers was used for PDSA cycle 4 to examine further if improvements could be continued and sustained.

The quality improvement team completed four plan-do-study-act cycles (PDSA) cycles. Patients age 0 to 16 years old received general anesthesia with a cuffed ETT. We did not include patients with known airway abnormalities. Patients presenting for emergency surgery were excluded. The anesthesia team administered premedication and performed induction at their discretion. We calculated the ETT size using the Duracher formula (ETT size = age in years/4 + 3.5) to remove variation in ETT sizing among providers. Patients were excluded if the ETT size used clinically deviated from the calculated size using the Duracher formula although this did not occur. Due to the infrequent use of a cuff manometer in clinical practice, the QI team focused on interventions that would be practical in the clinical setting.^19–21^

Throughout the QI initiative, we measured cuff pressures using a pressure transducer or a Posey cuff manometer. When using the pressure transducer, a stop cock was attached to allow for adding or removing air from the ETT cuff (**see picture, Supplemental Digital Content 1,** http://links.lww.com/PQ9/A439). The cuff pressure was measured in mm Hg and converted to cm H_2_O using a standard conversion ratio (0.76 mm Hg = 1 cm H_2_O). In PDSA cycle 2, the anesthesia team inserted a CARESCAPE Pedi-lite+ spirometry unit (GE Healthcare, Chicago, Ill.) between the 15-mm adaptor of the ETT and the anesthesia circuit for accurate titration of inspiratory and expiratory tidal volumes (TVs).

## STUDY OF THE INTERVENTION

### PDSA Cycle 1: Addition of Air

PDSA cycle 1 tested a stethoscope-guided inflation method by adding air. This technique is considered an “estimation” method. After confirming ETT placement, a member of the QI project team placed a stethoscope on the patient’s neck in the suprasternal notch over the trachea. Then, with a syringe attached to the pilot balloon, the provider added air to the ETT cuff until a leak was no longer audible while holding continuous airway pressure at 20 cm H_2_O. At this point, the cuff pressure was measured.

### PDSA Cycle 2: TV Ratio

A key driver identified was objective measurements. PDSA cycle 2 examined an inflation technique using inspired and expired TVs to inflate the ETT cuff objectively. This technique has been used in the literature as an alternative to ETT cuff pressure inflation.^27–29^ During this cycle, the team used a GE Pedi-lite spirometer to obtain the most accurate TV. They attached a pressure transducer with a stopcock to the pilot balloon of the ETT and zeroed at the mid-axillary line. This technique allowed for air insertion and removal as well as cuff pressure measurement. After ETT intubation, the cuff was inflated using the method of choice for the provider involved in patient care. Next, the team administered three lung recruitment maneuvers. This effort was completed by closing the adjustable pressure-limiting valve on the ventilator to 30 cm H_2_O and holding a breath for 10 seconds using the reservoir bag. Next, we placed the patient on volume-guaranteed mode to achieve a TV of 10 mL/kg, with a positive end-expiratory pressure of 5 cm H_2_O. Finally, we adjusted the respiratory rate to maintain normocarbia. The QI project team measured a baseline cuff pressure with the pressure transducer. Next, the air was removed in small increments observing the spirometry. Cuff pressures were recorded when inspired and expired TVs were equal.

### PDSA Cycle 3: Removal of Air

We examined a stethoscope-guided inflation method with air removal for PDSA cycle 3. This technique is considered another “estimation” method. After ETT placement, a member of the QI project slightly overinflated the cuff to seal the airway. Next, a stethoscope was placed on the patient’s neck in the suprasternal notch over the trachea. Then, the air was removed from the cuff with a syringe in small increments until auscultation of an air leak while holding continuous positive airway pressure at 20 cm H_2_O. Then, we measured the cuff pressure.

### PDSA Cycle 4: Further Testing of the Removal of Air Method

PDSA cycle 4 continued to examine the removal of air method. As this technique produced the most accurate and reliable results among the PDSA cycles, the QI team decided to further examine and assess for continued improvement. PDSA cycle 4 was 5 weeks in length and consisted of 10 subjects each week. In addition, we included anesthesia personnel outside the quality improvement team allowing the inflation method to be tested among a larger group of providers.

Before the patient arrived in the operating room, the QI team performed a walk-through of the inflation process to ensure the technique’s comfort, understanding, and accuracy. After ETT intubation, the cuff was slightly overinflated. Air was removed until a leak was auscultated with a stethoscope over the patient’s trachea, holding a continuous airway pressure at 20 cm H_2_O. At this point, we measured cuff pressures.

### Measures and Analysis

The primary outcome measure was the percentage of ETT cuff pressures within the recommended range of 20–30 cm H_2_O. We calculated the median and interquartile range (IQR) or mean and standard deviation (SD) for numerical variables and number (N) and percentages for categorical variables. We calculated differences between the groups using analysis of variance (ANOVA). We used descriptive statistics to assess trends. Data were collected using Microsoft Excel (Microsoft Corporation, Redmond, Va.) and analyzed using SAS 9.4 (Cary, N.C.). The project team collected the patient’s age, weight, ETT size, and cuff pressure for each subject. We used a statistical process control p-chart to measure the impact of our interventions over time with control limits set at ±3 SDs. A *P* value of <0.05 was considered statistically significant.

### Ethical Considerations

This initiative was deemed a quality improvement by the University of Cincinnati and Nationwide Children’s Hospital Institutional Review Boards and required no additional review.

## RESULTS

In total, we measured 150 cuff pressures. The median age of patients included was 6 years old, with a median weight of 24 kg (**see Table, Supplemental Digital Content 2,** http://links.lww.com/PQ9/A440). Cuffed ETT sizes varied as patient ages varied (**see Table, Supplemental Digital Content 3,** http://links.lww.com/PQ9/A441).

### Preimplementation Data

The quality improvement team collected preimplementation data on 25 patients to establish baseline ETT cuff pressures at Nationwide Children’s Hospital. The mean ETT cuff pressure was 36 ± 23 cm H_2_O with a range of 0 to 98 cm H_2_O. Cuff pressures were out of the patients’ recommended range in 76% (n = 19). Most ETT cuffs were overinflated as cuff pressures were higher than 30 cm H_2_O 52% (n = 13).

### PDSA Cycle 1: Addition of Air

PDSA cycle 1 consisted of a stethoscope-guided inflation method with the addition of air until a leak was no longer auscultated. The cohort consisted of 25 participants. The mean cuff pressure was 33 ± 14 cm H_2_O, ranging from 8 to 64 cm H_2_O. Cuff pressures were out of the recommended range 64% (n = 16) of the time.

### PDSA Cycle 2: TV Ratio

PDSA cycle 2 examined inspired and expired TVs to guide the ETT cuff inflation. The mean cuff pressure in the 1.0 ratio (expired:inspired TV = 1.0) was 14 ± 10 cm H_2_O ranging from 1 to 41 cm H_2_O. Cuff pressures were out of the recommended range 84% of the time (n = 23), with the majority of cuff pressures being below 20 cm H_2_O (n = 21).

### PDSA Cycle 3: Removal of Air

PDSA cycle 3 tested stethoscope-guided inflation with air removal. This cycle consisted of 25 patients with a mean cuff pressure of 20 ± 4 cm H_2_O with pressures ranging from 14 to 28 cm H_2_O. Cuff pressures were out of the recommended range 50% (n = 13) of the time. Therefore, no ETT cuffs were considered overinflated, as there were no cuff pressures above 30 cm H_2_O.

### PDSA Cycle 4: Further Testing of the Removal of Air Method

**Fig. 3.**
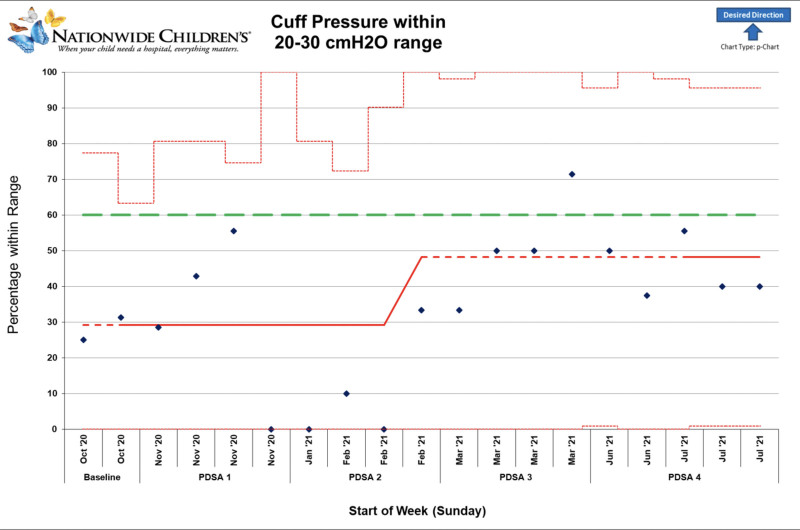
P chart examining the percentage of cuff pressures in the recommended range throughout PDSA cycles.

**Fig. 4.**
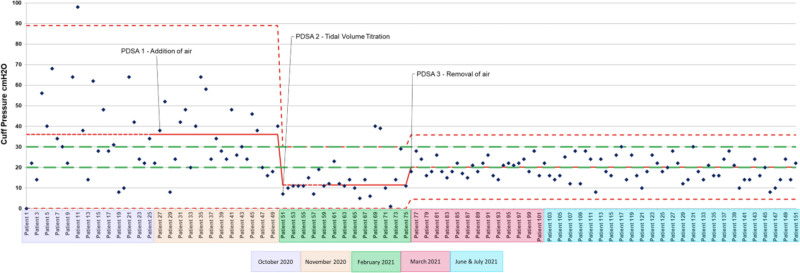
Control chart following measured cuff pressures throughout the quality improvement initiative.

PDSA 4 examined the removal of the air method for a longer time and among a larger group of anesthesia providers. Cuff pressures were out of the recommended range 54% of the time (n = 27), which was a statistically significant increase in the frequency of accurate measurements (*P* < 0.001) compared to the other methods examined. The mean cuff pressure was 19 ± 6 cm H_2_O, ranging from 8 to 30 cm H_2_O. Again, no cuff pressures were greater than 30 cm H_2_O. The removal of air method produced the most accurate and consistent cuff pressures (Table 1 and Figs. 3 and 4).

**Table 1.** Proportion of Cuff Pressures within Recommended Range by PDSA Cycle (Outcome Measure)

	Proportion of Cuff Pressures between 20 and 30 cm H_2_O
Preimplementation	6 of 25 (24%)
PDSA cycle 1	9 of 25 (36%)
PDSA cycle 2	2 of 25 (8%)
PDSA cycle 3	13 of 25 (52%)
PDSA cycle 4	23 of 50 (46%)

## DISCUSSION

### Summary

Using quality improvement science, we demonstrated an increase in the percentage of cuff pressure within the recommended range of 20–30 cm H_2_O. After completing a series of four PDSA cycles, our QI initiative established sustained and scaled improvements. We had the greatest improvements using the removal of air method. The leadership and anesthesia providers’ acceptance and willingness to develop change was a strength that provided a solid foundation for the initiative.

### Interpretation

ETT cuffs are often overinflated.^6–9,11^ This has been demonstrated in the literature and was also evident during our quality improvement initiative. It is worth noting that no cuff pressures were greater than 30 cm H_2_O in PDSA cycle 3 and 4. These cycles examined the removal of air technique. This method may improve patient care by decreasing the risk of overinflating the ETT cuff. We saw decreased cuff pressure dispersion as the project progressed (Fig. 4). The removal of the air method used to inflate ETT cuffs reduced the cuff pressure variation. This decrease in variation demonstrates that the inflation methods examined are more reliable and consistent than previously used in the clinical setting.

There is no cost increase to perform the air technique removal, as each anesthesia provider already has a stethoscope and a syringe to inflate the ETT cuff. This easy and accurate modification to anesthesia practice does not require additional time, effort, or financial resources; therefore, it will likely succeed in the long term. As in our improvement project, certain estimation techniques, like the air removal method, may lead to more appropriate cuff pressures. Still, estimation methods may not be the best way to obtain optimal cuff pressures consistently. Ultimately, subjective measurements have limitations. Although improvements were made after the QI project implementation, the percentage of cuff pressures remaining out of the recommended range was still significant (50%) and could lead to preventable patient harm. When estimation techniques are being used, or objective measurements are not an option, the removal of air method may be suitable for the inflation of ETT cuffs. It is a quick, simple, and practical inflation method.

### Limitations

We conducted this QI initiative at a single institution and used a convenience sample. We found statistically significant differences in cuff pressure measurements between the techniques examined, but research is needed to determine the accuracy of each technique. As anesthetists became more aware of the project, a change in practice could have occurred since clinical behaviors may change when providers know they are being observed. Cuff pressures were only measured directly after ETT intubation. Cuff pressures are a dynamic measurement, and fluctuations can be seen with changes in positioning, neuromuscular relaxation, temperature, altitude, use of nitrous oxide, and positive pressure ventilation.^22^ Measurement just after ETT placement may not be reflective of pressures throughout intubation. There was also an increase in cost and setup associated with additional equipment (GE Pedi-lite spirometer, Posey cuff manometer, pressure transducer). When using a cuff manometer, a small air leak caused by the direct measurement can occur as the manometer opens the one-way valve in the pilot balloon.^13,22,23,26^ This occurred during the project and was more problematic in patients younger than 1 year of age, resulting in difficulty achieving adequate ventilation after measuring the cuff pressure. This limitation is frequently observed in the clinical setting using a cuff manometer.^13,22,23,30,31^

### Next Steps and Recommendations

Although the improvements did not necessarily meet our goal, it is a positive and sustained impact on our cuff pressures. We hope that more widespread practice and use will lead to further improvements. Decreasing the variation in cuff inflation methods used in the clinical setting should be a focus. If estimation methods continue to be used most often in the clinical setting, examining which ones are most accurate is essential. As there is no standard practice, anesthesia providers’ use of many different methods may contribute to our inaccuracies. Ultimately, anesthesia providers need an accurate and reliable way to inflate the ETT cuff while not significantly increasing the time and cost needed for patient care. Systems will not see positive change if no appropriate or reasonable practice alternatives exist. Developing an objective way to measure cuff pressures that is reasonable, simple, and accurate would be helpful. Further research examining objective methods is warranted.

### Conclusions

This project adds to the vast amount of literature demonstrating that current inflation methods produce an unacceptably high rate of cuff pressures outside the recommended range but also establishes appropriate data to support changes in clinical practice. These results establish that removing air until auscultation of an audible air leak may be an effective and practical tool for anesthesia providers to use for ETT cuff inflation. This technique is an easy change in practice that could lead to fewer patient complications related to ETT cuffs. The improvement team will continue to monitor cuff pressures in the operating room to assess for improvement or regression. The removal of air technique may be an accurate method that uses no additional equipment or time during induction. Improving the accuracy of ETT cuff pressures optimizes patient safety and enhances the quality of anesthesia care. More data are needed, but our quality improvement initiative improved practice.

## ACKNOWLEDGMENT

The authors thank Jenah Eastep for assisting with the quality improvement project.

## DISCLOSURE

The authors have no financial interest to declare in relation to the content of this article.

## Supplementary Material


