# Supplementary material for: Endotracheal Tube Cuff Pressures in the Operating Room of a Pediatric Hospital: A Quality Improvement Initiative
Source: Pediatr Qual Saf. 2022 Dec 7;7(6):e619. doi: 10.1097/pq9.0000000000000619 (PMC9742117; doi:10.1097/pq9.0000000000000619)

Supplemental Digital Content

Endotracheal Tube Cuff Pressures in the Operating Room of a Pediatric Hospital: A Quality Improvement Initiative

Kelly Moon

Supplemental Digital Content 1: Pressure transducer with stop cock attached for the continuous measurement of cuff pressure

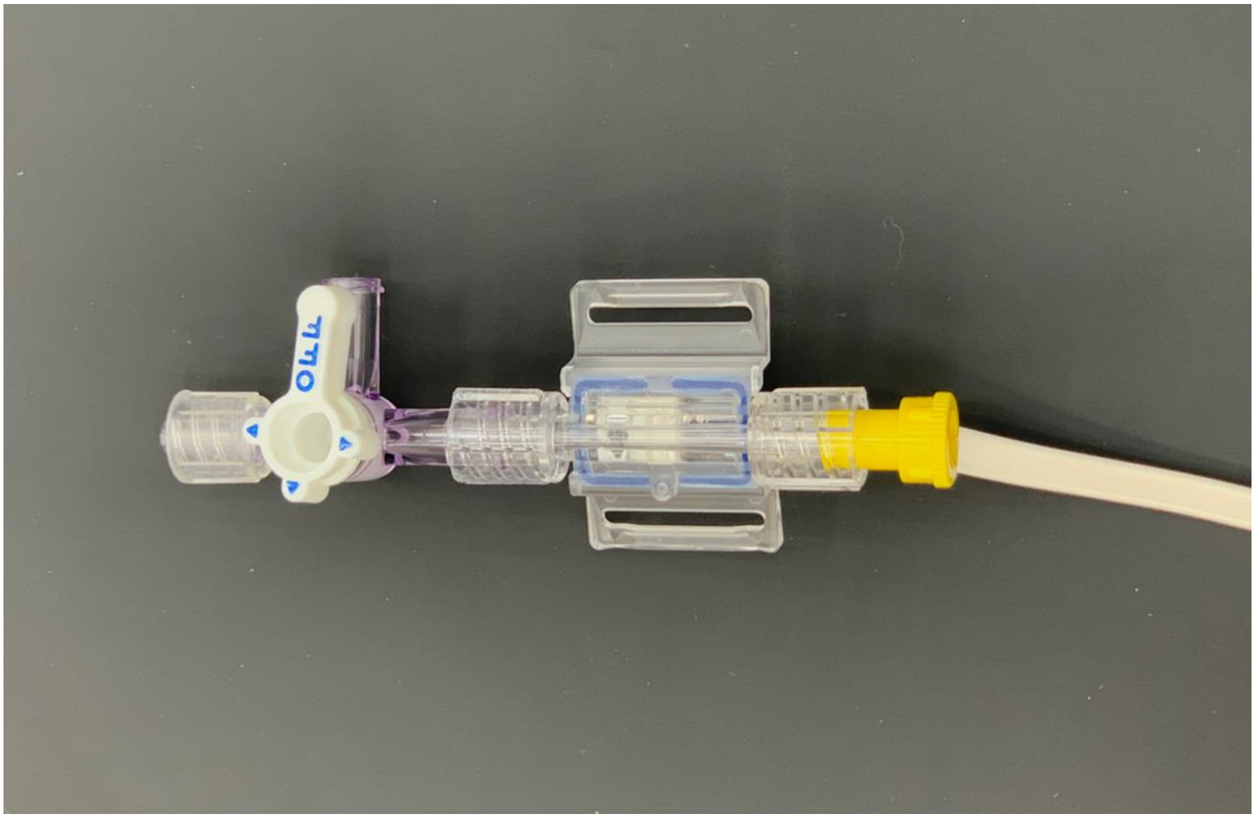

Supplement: Supplementary file 1 [file pqs-7-e619-s001.pdf]
