# Supplementary material for: Endotracheal Tube Cuff Pressures in the Operating Room of a Pediatric Hospital: A Quality Improvement Initiative
Source: Pediatr Qual Saf. 2022 Dec 7;7(6):e619. doi: 10.1097/pq9.0000000000000619 (PMC9742117; doi:10.1097/pq9.0000000000000619)
Supplement: Supplementary file 2 [file pqs-7-e619-s002.pdf]

<u>Supplemental Digital Content</u>

Endotracheal Tube Cuff Pressures in the Operating Room of a Pediatric Hospital: A Quality Improvement Initiative

Kelly Moon

Supplemental Digital Content 2: Table displaying patient demographics for the quality improvement initiative

	**All (*N* = 150)**	**Baseline (*n* = 25)**	**PDSA 1 Addition of Air (*n* = 25)**	**PDSA 2 Tidal Volume Titration (*n* = 25)**	**PDSA 3 Removal of Air (*n* = 25)**	**PDSA 4 Removal of Air (*n* = 50)**
**Age (years)**	6 (3, 12)	6 (3, 13)	6 (3, 13)	9 (4, 14)	7 (2, 11)	6 (3, 12)
< 1 year	11 (7%)	1 (4%)	1 (4%)	0	2 (8%)	7 (14%)
1-4	51 (34%)	11 (44%)	9 (36%)	9 (36%)	8 (32%)	14 (28%)
5-9	33 (22%)	2 (8%)	6 (24%)	4 (16%)	6 (24%)	15 (30%)
10-12	21 (14%)	4 (16%)	2 (8%)	4 (16%)	4 (16%)	7 (14%)
> 13	34 (23%)	7 (28%)	7 (28%)	8 (32%)	5 (20%)	7 (14%)
**Weight (kilograms)**	24 (15, 48)	29 (14, 51)	24 (17, 51)	35 (19, 58)	27 (13, 45)	20 (14, 38)
