# Supplementary material for: Endotracheal Tube Cuff Pressures in the Operating Room of a Pediatric Hospital: A Quality Improvement Initiative
Source: Pediatr Qual Saf. 2022 Dec 7;7(6):e619. doi: 10.1097/pq9.0000000000000619 (PMC9742117; doi:10.1097/pq9.0000000000000619)
Supplement: Supplementary file 3 [file pqs-7-e619-s003.pdf]

<u>**Supplemental Digital Content**</u>

Endotracheal Tube Cuff Pressures in the Operating Room of a Pediatric Hospital: A Quality Improvement Initiative

Kelly Moon

Supplemental Digital Content 3: Table that illustrates cuff endotracheal tube sizes used throughout the quality improvement project

Table 2: Cuffed Endotracheal Tube Size

**Endotracheal Tube Size (ID mm)**	**All (*N* = 100)**	**Baseline (*n* = 25)**	**PDSA 1 Addition of air (*n* = 25)**	**PDSA 2 Tidal Volume Titration (*n* = 25)**	**PDSA 3 Removal of Air (*n* = 25)**	**PDSA 4 Removal of Air (*n* = 50)**
3.0	8 (5%)	0	1 (4%)	0	2(8%)	5 (10%)
3.5	10 (7%)	4 (16%)	2 (8%)	0	1 (4%)	3 (6%)
4.0	24 (16%)	4 (16%)	5 (20%)	0	4 (16%)	11 (22%)
4.5	28 (19%)	4 (16%)	4 16%)	9 (36%)	4 (16%)	7 (14%)
5.0	16 (11%)	2 (8%)	2 (8%)	3 (12%)	3 (12%)	6 (12%)
5.5	11 (7%)	0	2 (8%)	1 (4%)	2 (8%)	6 (12%)
6.0	20 (13%)	6 (24%)	2 (8%)	3 12%	5 (20%)	4 (8%)
7.0	33 (22%)	5 (20%)	7 (28%)	9 (36%)	4 (16%)	8 (16%)
